# High fat diet altered cardiac metabolic gene profile in *Psammomys obesus* gerbils

**DOI:** 10.1186/s12944-020-01301-y

**Published:** 2020-06-03

**Authors:** Abdelhamid Sahraoui, Céline Dewachter, Grégory Vegh, Kathleen Mc Entee, Robert Naeije, Souhila Aouichat Bouguerra, Laurence Dewachter

**Affiliations:** 1 Laboratory of Physiology and Pharmacology, Faculty of Medicine, Université Libre de Bruxelles, 808, Lennik Road, 1070 Brussels, Belgium; 2 Team of Cellular and Molecular Physiopathology, Faculty of Biological Sciences, Houari Boumediene University of Sciences and Technology, El Alia, Algiers, Algeria; 3 Faculté des Sciences de la Nature et de la Vie & des Sciences de la Terre, University Djilali Bounaama of Khemis Miliana, 44225 Khemis Miliana, Algeria; 4 Department of Cardiology, Cliniques Universitaires de Bruxelles, Hôpital Académique Erasme, Bruxelles, Belgium

**Keywords:** Lipotoxicity, *Psammomys obesus*, High fat diet, Cardiomyopathy, Apoptosis

## Abstract

**Background:**

In metabolic disorders, myocardial fatty infiltration is critically associated with lipotoxic cardiomyopathy.

**Methods:**

Twenty *Psammomys obesus* gerbils were randomly assigned to normal plant or high fat diet. Sixteen weeks later, myocardium was sampled for pathobiological evaluation.

**Results:**

A sixteen-week high fat diet resulted in myocardial structure disorganization, with collagen deposits, lipid accumulation, cardiomyocyte apoptosis and inflammatory cell infiltration. Myocardial expressions of glucose transporter GLUT1 and pyruvate dehydrogenase (PDH) inhibitor, PDH kinase (PDK)4 increased, while insulin-regulated GLUT4 expression remained unchanged. Myocardial expressions of molecules regulating fatty acid transport, CD36 and fatty acid binding protein (FABP)3, were increased, while expression of rate-controlling fatty acid β-oxidation, carnitine palmitoyl transferase (CPT)1B decreased. Myocardial expression of AMP-activated protein kinase (AMPK), decreased, while expression of peroxisome proliferator activated receptors (PPAR)-α and -γ did not change.

**Conclusion:**

In high fat diet fed *Psammomys obesus*, an original experimental model of nutritionally induced metabolic syndrome mixing genetic predisposition and environment interactions, a short period of high fat feeding was sufficient to induce myocardial structural alterations, associated with altered myocardial metabolic gene expression in favor of lipid accumulation.

## Background

In the normal adult heart, fatty acids are major substrates for ATP generation, while glucose oxidation provides a lesser contribution to energy production [1]. Besides an altered metabolic profile, chronic high fat diet consumption contributes to dysregulated glucose and fatty acid metabolism, leading to overall dysregulated energy homeostasis [2]. In the heart, this is associated with a variety of adaptations and alterations in myocardial structure and function occurring in subjects as adipose tissue and lipids accumulate abnormally, even in the absence of comorbidities such as type 2 diabetes or hypertension [3]. Obesity has been associated with increased myocardial lipid accumulation [4, 5], which was correlated with diastolic dysfunction [6]. Increased myocardial intracellular levels of triglycerides were also commonly described in various experimental models of obesity [7, 8]. Chronic exposure to high plasma levels of free fatty acids may cause accumulation of toxic lipid intermediates within cardiomyocytes, which has been related to cardiac lipotoxicity [9]. However, mechanisms leading to obesity-induced cardiomyopathy remain largely unknown [10].

Due to its particular pathologic adaptation to nutrient excess, *Psammomys obesus* is an original nutritionally controlled and genetically predetermined experimental model of obesity and metabolic syndrome [11]. Indeed, on its natural diet composed of halophilic plants, *Psammomys obesus* is healthy, with a metabolic-endocrine system adapted to desert life. In captivity, they spontaneously and naturally develop diabetes, dyslipidemia and obesity, when fed on a standard laboratory rodent chow diet [11]. This pathologic adaptation to nutrient excess may represent a reliable experimental model for studying the mechanisms underlying the predisposition to develop insulin resistance and metabolic syndrome in humans who evolve from scarcity to abundant food intake [12]. In these animals, a short period of time with high fat diet resulted in an important weight gain and increased circulating levels of fatty acids, as well as altered myocardial expression of calcium-handling contraction proteins [13].

In this context, the objective of the present study was to explore whether a high fat diet could induce cardiac alterations in structure, energy metabolism and cardiomyocyte viability in these wild rodents with a genetic predisposition to develop obesity.

## Methods

### Animal model

Adult *Psammomys obesus* gerbils were captured in the Algerian region of Beni-Abbes (30°7 latitude north and 2°10 longitude west) and housed in individual cages in a 12-h light/dark cycle for 2 weeks. During this period of acclimation, the animals were fed with their natural food composed of halophilic plants [14, 15]. Thereafter, sex-matched eight-week old gerbils weighting 93 ± 9 g were divided in 2 groups as follows: 1) control animals were fed with normal diet of natural halophilic plants (*Salicornia*; composition of the halophilic plants: water 80.8 g; mineral salts 6.9 g; lipids 0.4 g; proteins 3 g; carbohydrates 8.4 g and 45–50 kcal/100 g); 2) the other group of animals received a high fat diet, comprising halophilic plants plus the daily addition of one-quarter (5 g) of cooked egg yolk (composition of cooked egg yolk: water 40–46 g; proteins 13.5–17.5 g; carbohydrates 0.2 g; lipids 30–31 g; cholesterol 1.2–1.3 g and 370–400 kcal/ 100 g) during 16 weeks.

At baseline and after a sixteen-week high fat diet, animals were bled from the retro-orbital venous plexus. Blood samples were immediately centrifuged at 3000 rpm on dried tubes. At the end of the protocol, the animals were sacrificed by decapitation. Hearts were immediately dissected, snap-frozen in liquid nitrogen and kept at − 80 °C for pathobiological analysis (*n* = 10 in each group) or after three-day fixation in Bouin’s aqueous solution, embedded in toto in paraffin for histopathological evaluation (*n* = 10 in each group).

### Biochemical analysis

Glucose, triglyceride and total cholesterol concentrations were determined with BIOSYSTEM kits (Barcelona, Spain) in plasma samples, according to manufacturer’s instructions. Plasma were used for the assay of lipoproteins on agarose gel by the method of Kalwakami [16]. Plasma creatine Phosphokinase (CPK) levels were measured using COBAS INTEGRA Analyzer (Roche; CA, USA).

### Cardiac morphometry

Five-micrometer myocardial sections were taken along the longitudinal axis of the heart and stained with hematoxyllin-eosin for overall morphological analysis, as previously described [17]. Masson’s Trichrome staining was used to assess collagen accumulation and fibrosis within myocardial sections.

### Immunohistochemistry: Detection of myocardial cells undergoing apoptosis

Cardiac apoptotic cells were detected by Terminal Deoxynucleotidyl Transferase dUTP Nick-End Labeling (TUNEL) staining using the *ApopTagPlus Peroxidase* In Situ *Apoptosis Detection Kit* (Chemicon, Temecula, CA), according to the manufacturer’s instructions. Negative control run without TdT enzyme and positive control pretreated with DNase-I were tested. For each cardiac sample, ten different randomly chosen fields were examined. Cardiac apoptotic rate was calculated as the ratio of apoptotic nuclei (TUNEL-positive or brown nuclei) to total nuclei (brown + blue nuclei) (× 100 to be expressed in percentage). All counts were performed by two independent investigators in blinded manner. Mean value was used for analysis.

### Real–time quantitative polymerase chain reaction (RTQ-PCR)

Total RNA was extracted from snap-frozen myocardial tissue using QIAGEN RNeasy®Mini kit (QIAGEN, Hilden, Germany), according to the manufacturer’s instructions. RNA concentration was determined by standard spectrophotometric technique and RNA integrity was assessed by visual inspection of GelRed (Biotium, Hayward, California)-stained agarose gels. Reverse transcription was performed using random hexamer primers and Superscript II Reverse Transcriptase (Invitrogen, Merelbeke, Belgium), according to the manufacturer’s instructions.

For RTQ-PCR, sense and antisense primers (Table 1) were designed using Primer3 program for *Rattus norvegicus* solute carrier family 2 members 1 (Slc2a1 or GLUT1) and 4 (Slc2a4 or GLUT4), carnitine palmitoyltransferase1B (CPT1B), fatty acid translocase CD36, fatty acid binding protein 3 (FABP3), peroxisome proliferator activated receptors (PPAR) -alpha and -gamma, AMP-activated protein kinase (AMPK), pyruvate dehydrogenase kinase (PDK) 4, uncoupling protein (UCP) 3, insulin receptor substrates (IRS) 1 and 2, natriuretic peptide B (NPPB) and hypoxanthine phosphoribosyl transferase (HPRT) 1 mRNA sequences. To avoid inappropriate amplification of residual genomic DNA, intron-spanning primers were selected when exon sequences were known. For each sample, amplification reaction was performed in triplicate using SYBR Green PCR Master Mix (Quanta Biosciences, Gaithersburg, MD, USA), specific primers and diluted template cDNA. Result analysis was performed using an iCycler System (BioRad Laboratories). Relative quantification was achieved with the comparative 2^-ΔΔCt^ method by normalization with the housekeeping gene (HPRT1). Results were expressed as relative fold increase over the mean value of relative mRNA expression of 16-week normal diet fed control group arbitrary fixed to 1.

**Table 1:** Primers used for real-time quantitative polymerase chain reaction (RT-QPCR) in myocardial tissue from *Psammomys obesus* gerbils

Genes		Primer sequences
Solute carrier family 2 members 1 (**Slc2a1 **or **GLUT1**)	*Sense*	GTGGGCCTCTTTGTTAATCG
	*Antisense*	CATAAGCACGGCAGACACAA
Solute carrier family 2 members 4 (**Slc2a4 **or **GLUT4**)	*Sense*	GAGTTAGCTGGGGTGGAACA
	*Antisense*	ACCGAGACCAACGTGAAGAC
Carnitine palmitoyltransferase-1B (**CPT1B**)	*Sense*	AAGAACACGAGCCAACAAGC
	*Antisense*	ACCATACCCAGTGCCATCAC
Fatty acid binding protein 3 (**FABP3**)	*Sense*	TCAAGTCGGTCGTGACACTG
	*Antisense*	TCCCATCACTTAGTTCCCGTG
Fatty acid translocase (**CD36**)	*Sense*	TTCAAGGTGTGCTCAACAGC
	*Antisense*	ACCCCACAAGAGTTCCTTCA
AMP-activated protein kinase (**AMPK**)	*Sense*	TTCGGGAAAGTGAAGGTGGG
	*Antisense*	TCTCTGCGGATTTTCCCGAC
Pyruvate dehydrogenase kinase 4 (**PDK4**)	*Sense*	TTCCAGGCCAACCAATCCAC
	*Antisense*	TGGCCCTCATGGCATTCTTG
Uncoupling protein 3 (**UCP3**)	*Sense*	CGCCAGATGAGTTTTGCCTC
	*Antisense*	CTGGAGTGGTCCGTTCCTTT
Peroxisome proliferator activated receptor-alpha (**PPAR-α**)	*Sense*	TTAGAGGCGAGCCAAGACTG
	*Antisense*	CAGAGCACCAATCTGTGATGA
Peroxisome proliferator activated receptor-gamma (**PPAR-γ**)	*Sense*	GCGCTAAATTCATCTTAACTCCCA
	*Antisense*	CTGTGTCAACCATGGTAATTTCAGT
Insulin receptor substrate 1 (**IRS1**)	*Sense*	ATGAACATCAGACGCTGTGG
	*Antisense*	TCATCCACTTGCATCCAGAA
Insulin receptor substrate 2 (**IRS2**)	*Sense*	CGGATTTTGGAAGAGGAGAGA
	*Antisense*	GAGTGATGAGGCTGGGTATGA
Natriuretic peptide B (**NPPB**)	*Sense*	GACGGGCTGAGGTTGTTTTA
	*Antisense*	ACTTGAGAGGTGGTCCCAGA
Hypoxanthine phosphoribosyl transferase-1 (**HPRT1**)	*Sense*	ACAGGCCAGACTTTGTTGGA
	*Antisense*	TCCACTTTCGCTGATGACCAC

### Statistical analysis

All data were expressed as mean ± standard error of the mean (SEM). Statistical analyses were performed using StatView 5.0 Software. Intergroup differences were assessed by one-way analysis of variance (one-way ANOVA) followed by Student’s t-test. *p* < 0.05 was considered statistically significant.

## Results

### Chronic high fat diet caused body weight gain and systemic hyperlipidemia

As illustrated in Table 2, baseline body weight and biochemical parameters were similar between the two study groups of *Psammomys obesus* gerbils. After sixteen-week high fat diet, body weight increased more than in normal diet-fed animals (Table 2).

**Table 2:** Sixteen-week evolution of bodyweight and plasma biochemical parameters after high fat and normal diet feeding in *Psammomys obesus* gerbils

	Baseline			After 16 weeks		
	Normal diet (***n*** = 10)	Hight fat diet (***n*** = 10)		Normal diet (***n*** = 10)	High fat diet (***n*** = 10)	
**Bodyweight (in g)**	91 ± 3	94 ± 2	**NS**	106 ± 5	113 ± 4	*
**Glucose (in g/L)**	0.62 ± 0.06	0.59 ± 0.03	**NS**	0.60 ± 0.07	0.79 ± 0.10	NS
**Triglycerides (in g/L)**	0.58 ± 0.05	0.64 ± 0.05	**NS**	0.52 ± 0.06	0.97 ± 0.16	*
**Total cholesterol (in g/L)**	0.61 ± 0.09	0.55 ± 0.03	**NS**	0.70 ± 0.08	4.29 ± 0.98	*
**LDL-cholesterol (ing/L)**	0.12 ± 0.03	0.12 ± 0.01	**NS**	0.15 ± 0.04	3.25 ± 0.83	**
**HDL-cholesterol (in g/L)**	0.35 ± 0.06	0.34 ± 0.02	**NS**	0.46 ± 0.05	1.06 ± 0.12	**
**Castelli risk index-I**	1.90 ± 0.45	1.64 ± 0.06	**NS**	1.87 ± 0.37	3.83 ± 0.76	*
**Castelli risk index-II**	0.69 ± 0.47	0.35 ± 0.04	**NS**	0.31 ± 0.05	2.72 ± 0.63	*
**CPK (IU/L)**	375 ± 42	361 ± 55	**NS**	345 ± 41	646 ± 106	*

Values are presented as means ± SEM. ** 0.001 < *P* < 0.05, *0.01 < *P* < 0.05 high fat versus normal diet feeding at baseline and after 16-week feeding. *LDL* means low-density lipoprotein, *HDL* high-density lipoprotein; Castelli risk index-I calculated as the ratio between total cholesterol and HDL-cholesterol levels; Castelli risk index-II calculated as the ratio between LDL-cholesterol and HDL-cholesterol levels; *CPK* creatine phosphokinase, *NS* not significant

In terms of biochemical parameters, sixteen-week high fat diet was associated with increased plasma levels of lipids, including triglycerides, total cholesterol, low-density (LDL-c) and high-density lipoprotein cholesterol (HDL-c) (Table 2). Plasma atherogenic Castelli’s Risk Index-I (assessed as the ratio between total cholesterol and HDL-cholesterol) and -II (assessed as the ratio between LDL-cholesterol and HDL-cholesterol) were calculated. They were found to be significantly increased after sixteen-week high fat diet (Table 2). There was only a trend (but not significant) of higher plasma level of glucose (increased by 32% compared to normal diet) (Table 2). Plasma levels of CPK were increased (Table 2).

### Myocardial architecture was altered after sixteen-week high fat diet

**Fig. 1:**
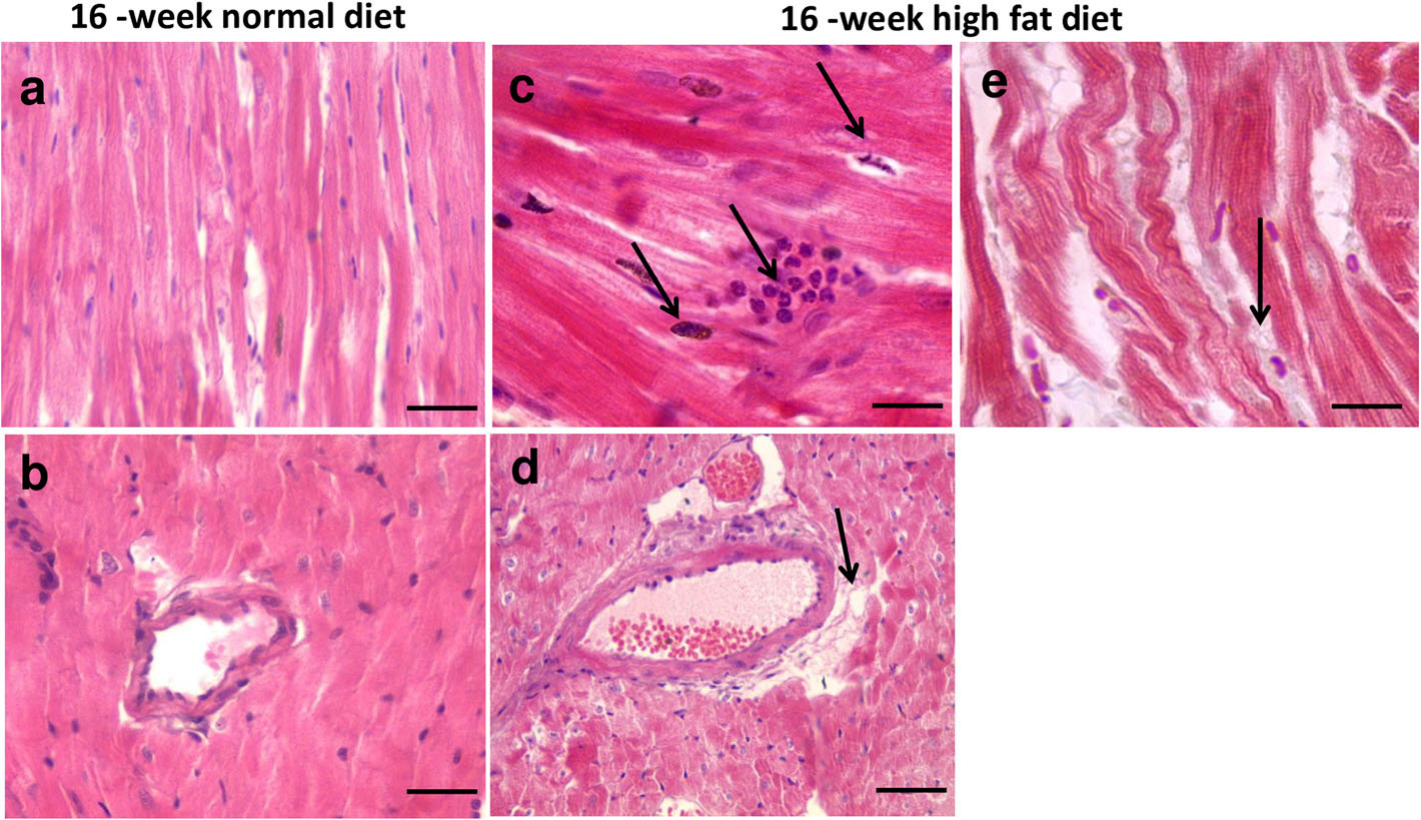
Representative hematoxylin and eosin-stained myocardial sections from *Psammomys obesus* gerbils fed with normal diet (**a**, **b**) or high fat diet (**c-e**) during 16 weeks. Myocardial sections were obtained at 1000-fold (**a**-**d**; Scale bars: 20 μm) and 400-fold (**e**; Scale bar: 50 μm) magnification. In normal diet-fed animals, myocardial sections showed normal histological heart architecture with myofibrils and muscle bundles (**a**, **b**). In 16-week high fat diet fed animals, hematoxylin and eosin stained-myocardial sections showed infiltrating inflammatory cells (**c**) and lipid accumulation (**d**, **e**)

As illustrated in Fig. 1a and b, hematoxylin and eosin staining showed normal histological heart architecture with myofibrils and muscle bundles in myocardial sections from normal diet fed animals. After sixteen-week high fat diet, there was myocardial accumulation of infiltrating inflammatory cells and apoptosis of cardiac myocytes (Fig. 1c). This was associated with lipid deposit accumulation within the myocardium (Fig. 1d and e), which strongly suggest myocardial lipotoxicity probably contributing to depressed cardiac function and cardiomyopathy [18].

**Fig. 2:**
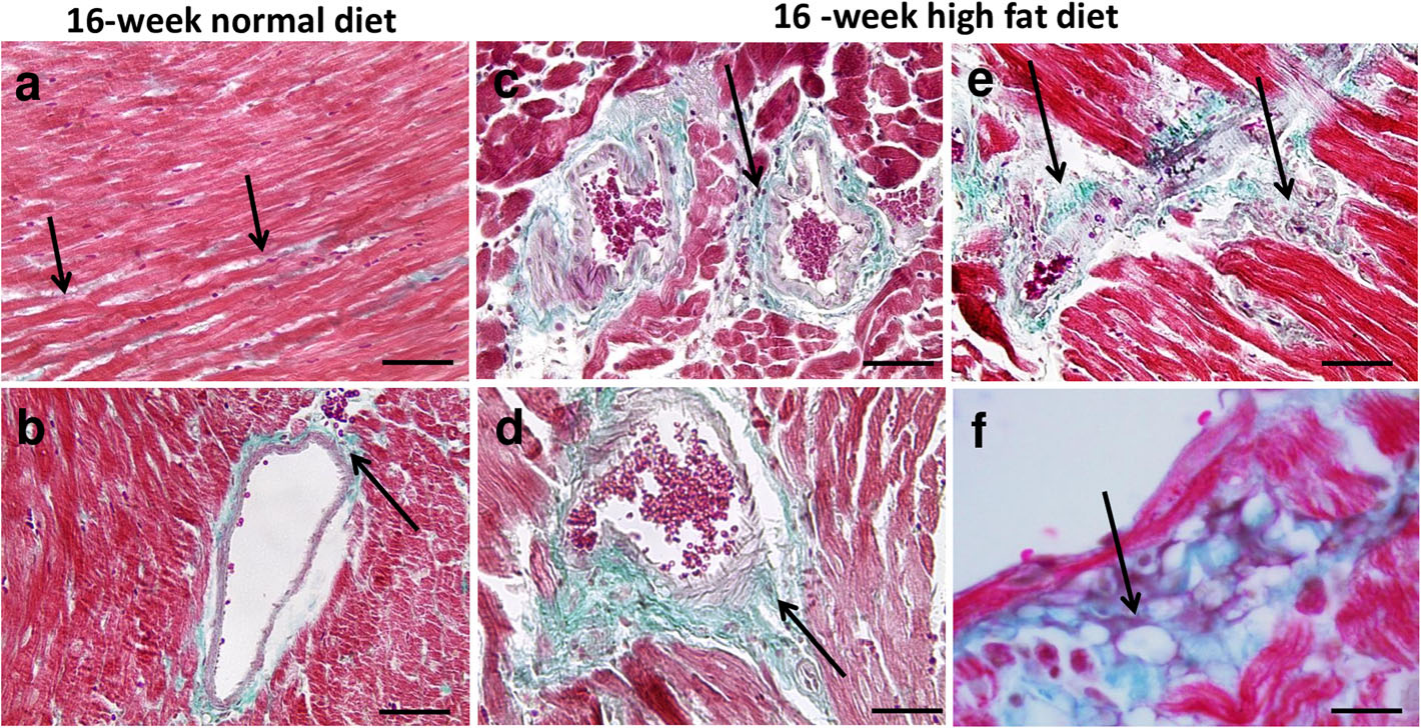
Representative Masson Trichrome-stained myocardial sections from *Psammomys obesus* gerbils fed with normal diet (**a**, **b**) or with high fat diet (**c-f**) during 16 weeks. Trichrome Masson staining was performed to detect fibrotic areas (collagen fibers stained in green; indicated by arrows in **a**-**e**). Myocardial sections were obtained at 400-fold (**a-e;** scale bars: 50 μm) and 1000-fold (**f**; scale bar: 20 μm) magnification. In animals fed with normal diet, cardiac cells were arranged orderly, with structured cardiac muscle fiber organization and little collagen fiber deposit (**a**, **b**). In 16-week high fat diet fed animals, Trichrome Masson-stained myocardial sections showed diffuse fibrosis (mostly in perivascular areas; **c** and **d**) and lipid deposit (**f**)

To assess fibrosis and collagen deposit, Masson’s Trichrome staining was performed. In animals fed with normal diet, cardiac cells were arranged orderly, with a structured cardiac muscle fiber organization and little collagen fiber deposit even at perivascular area (Fig. 2a and b). Sixteen-week high fat diet induced myocardial structural disorganization with cardiomyocyte loss (Fig. 2c and e), myocardial diffuse interstitial (Fig. 2e) and perivascular fibrosis (Fig. 2c and d), associated with myocardial accumulation of infiltrating cells (Fig. 2e) and lipid deposits (Fig. 2f).

### Cardiomyocyte apoptosis was induced by sixteen-week high fat diet

**Fig. 3:**
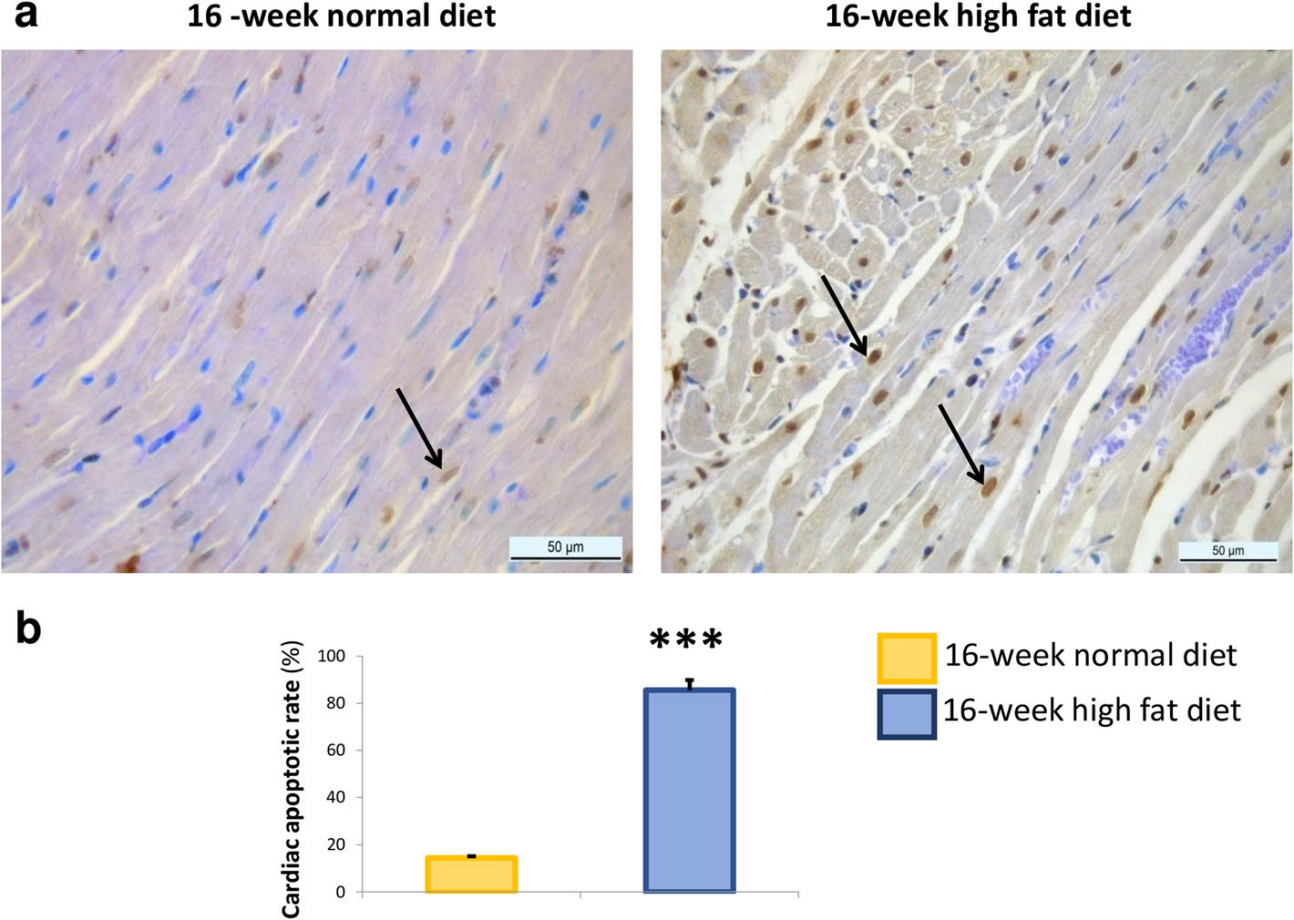
Myocardial activation of apoptotic processes assessed by terminal deoxynucleotidyl transferase biotin-dUTP nick-end labeling (TUNEL) in *Psammomys obesus* gerbils fed with normal diet or high fat diet during 16 weeks. Myocardial sections were obtained at 400-fold (**a**; Scale bars: 50 μm). Cardiac apoptotic rate (in percentage; **b**) was evaluated as the ratio between the number of terminal deoxynucleotidyl transferase biotin-dUTP nick-end labeling-positive cardiomyocytes (brown nuclei mentioned by arrows) and the total number of cardiomyocytes (brown + blue nuclei) in 16-week normal (*n* = 10; yellow bars) and high fat diet (*n* = 10; blue bars)-fed animals. Data are expressed as mean ± SEM.***P < 0.001 versus the normal diet fed animals

To assess whether this myocardial accumulation of lipids could be associated with cardiac cell apoptosis, a TUNEL staining was performed in myocardial sections. As illustrated in Fig. 3a, sixteen-week high fat diet induced diffuse apoptosis in cardiomyocytes. The apoptotic rate was increased in the myocardium of *Psammomys obesus* fed with high fat diet (Fig. 3b).

### High fat diet altered myocardial expression of molecules implicated in glucose and lipid metabolism

**Fig. 4:**
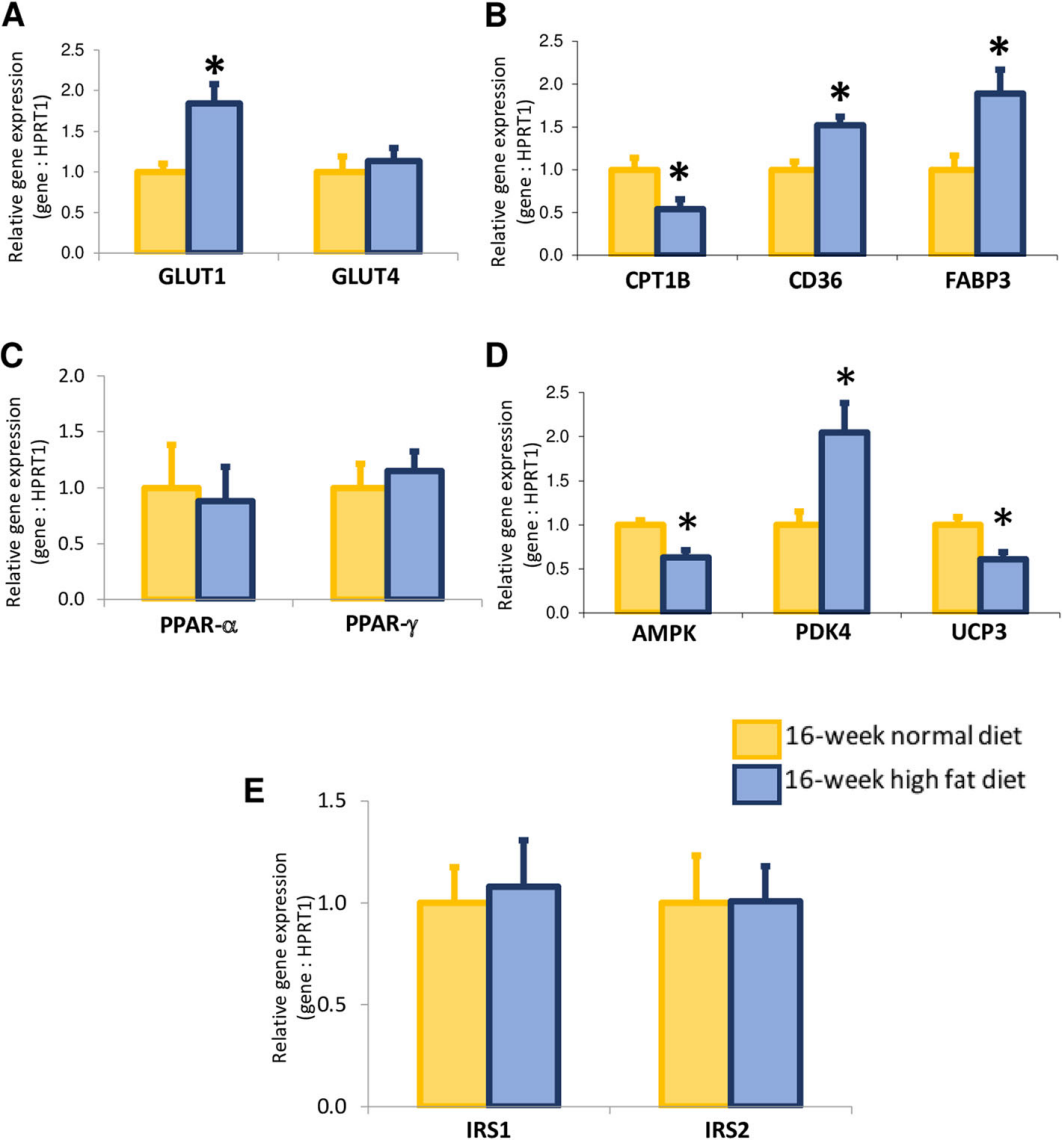
Myocardial expression of genes implicated in uptake and metabolism of glucose and fatty acids, including the solute carrier family 2 members 1 (Slc2a1 also called GLUT1; **a**) and 4 (Slc2a4 also called GLUT4; **a**); the carnitine palmitoyltransferase1B (CPT1B; **b**), the CD36 (**b**) and the fatty acid binding protein 3 (FABP3; **b**); of transcription factors implicated in cardiac metabolism regulation, including the peroxisome proliferator activated receptors-alpha (PPAR-α; **c**) and -gamma (PPAR-γ; **c**); the protein kinase AMP-activated (AMPK; **d**), pyruvate dehydrogenase kinase 4 (PDK4; **d**) and uncoupling protein 3 (UCP3; **d**); and of insulin receptors (IRS)1 and 2 (**e**) in 16-week normal (*n* = 10; yellow bars) versus high fat (*n* = 10; blue bars) diet fed *Psammomys obesus* gerbils. Values are expressed as mean ± SEM. * 0.01 < *P* < 0.05, high fat diet- versus normal diet-fed *Psammomys obesus* animals

To determine the effects of a relative short period of high fat diet on expression profile of genes modulating cardiac energy production, including cardiac glucose and fatty acid metabolism, as well as insulin signaling, RT-QPCR experiments were performed. Sixteen-week high fat diet increased myocardial gene expression of the major glucose transporter GLUT1, while expression of GLUT4, an insulin-regulated facilitative glucose transporter, remained unchanged (Fig. 4a). Myocardial expression of CD36, a major cellular regulator of fatty acid transport, and FABP3, an intracellular fatty acid-binding protein participating in fatty acid uptake and intracellular transport, were increased, while expression of the rate-controlling enzyme of fatty acid β-oxidation pathway, CPT1B, decreased (Fig. 4b). All these results suggest altered myocardial expression profile of energy production mediators in favor of myocardial fatty acid uptake and accumulation (decreased fatty acid β-oxidation) after sixteen-week high fat diet in *Psammomys obesus* gerbils.

To understand better mechanisms controlling the expression of these metabolic mediators, expression of nuclear transcription factors and enzymes implicated in lipid and glucose metabolism regulation and adipocyte differentiation were evaluated. As illustrated in Fig. 4c, myocardial expression of PPAR-α and -γ did not change after chronic high fat diet. However, expressions of AMPK, a cellular energy sensor, and of UCP3, a mitochondrial transporter implicated in energy balance control, decreased in the heart of animals fed with high fat diet, while expression of PDK4, which phosphorylates (and thus deactivates) pyruvate dehydrogenase (PDH) and inhibits glucose oxidation, was increased (Fig. 4d). Myocardial expressions of IRS1 and 2 did not change (Fig. 4e).

### Myocardial expression of wall stretch responsive gene, the natriuretic peptide B (BNP)

**Fig. 5:**
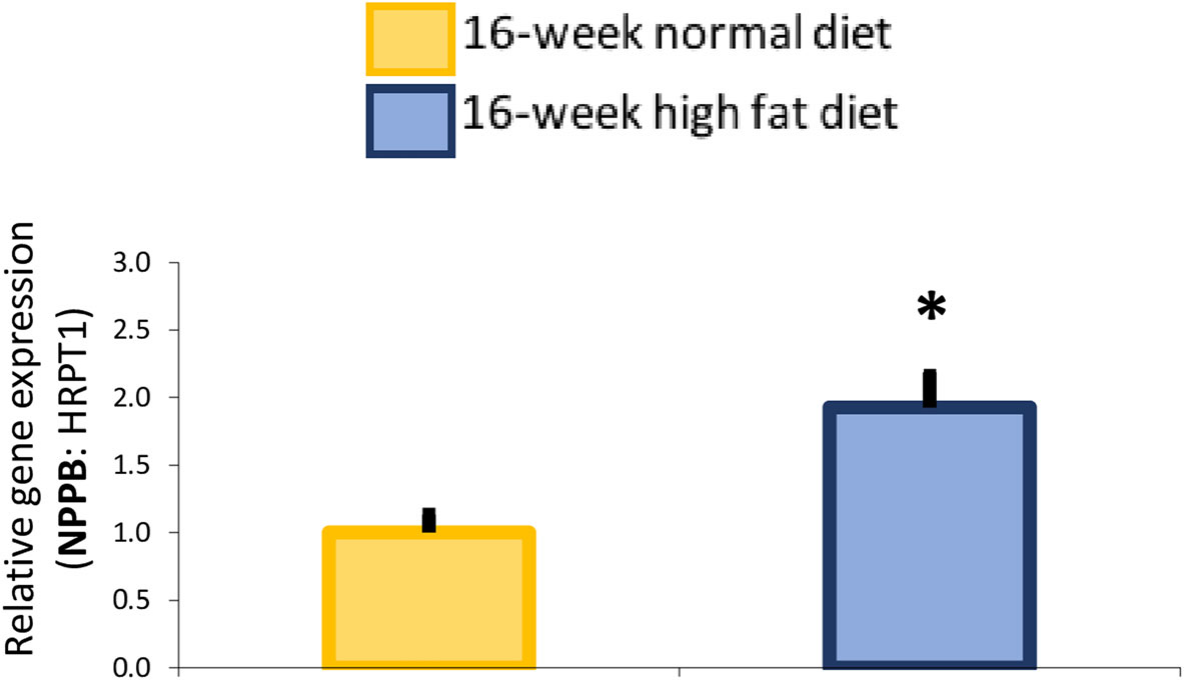
Myocardial expression of cardiac stress-response gene, natriuretic peptide B (NPPB) in 16-week normal (*n* = 10; yellow bars) versus high fat (*n* = 10; blue bars) diet fed *Psammomys obesus* gerbils. Values are expressed as mean ± SEM. * 0.01 < *P* < 0.05, high fat diet- versus normal diet-fed *Psammomys obesus* animals

When the heart is stretched, BNP concentration increases markedly, indicating that the heart is working harder and having more trouble meeting the body’s demands, such as in heart failure [19]. In the present study, gene expression of NPPB, the precursor of BNP, was increased in the myocardium of high fat diet-fed animals (Fig. 5).

## Discussion

The present results show that sixteen-week high fat diet results in severe alterations of cardiac structure, with collagen and lipid accumulation and diffuse activation of apoptotic processes in *Psammomys obesus* gerbils, wild rodents with a genetic predisposition to develop obesity, diabetes and metabolic syndrome. This was associated with altered cardiac expression of molecules regulating energy metabolism in favor of cardiac lipid accumulation, with increased expressions of CD36 and FABP3, both implicated in cellular fatty acid transport, and of PDK4, a key regulator of glucose oxidation, while expressions of CPT1B, a rate-controlling enzyme of fatty acid β-oxidation and of a key cellular energy sensor AMPK decreased.

A sixteen-week high fat diet resulted in the development of obesity associated with increased circulating levels of triglycerides, total cholesterol, LDL- and HDL-cholesterol, which strongly suggest major cardiovascular risks in these animals [20, 21]. This was confirmed by higher values of Castelli’s risk index-I and –II after sixteen-week high fat diet [22]. This is consistent with previous studies showing rapid development of obesity and metabolic syndrome after high fat diet in *Psammomys obesus* [13, 14, 23], even if a characteristic diabetic profile was not found in the present study. Indeed, glucose tolerance seemed to be preserved in these animals, because glucose levels only slightly increased. This apparent discrepancy has already been described in *Psammomys obesus* removed from their natural halophilic plant diet evolving variably overtime [15]. Genetic background also probably contributed to the rapid cardiac alterations in cardiac morphometry and energy substrate use. However, little is known about these genetic determinants. Genome sequencing of *Psammomys obesus* revealed a mutationally biased chromosome region probably involved in ecological adaptation and constraint in these animals [16]. However, to date, the link with the present results remain elusive.

After high fat diet, histological examination of the heart revealed interstitial and perivascular fibrosis and inflammatory cell infiltration, associated with lipid accumulation and cardiomyocyte apoptosis. In humans, obesity has been related to epicardial fat deposition and intra-myocardial fatty infiltration [17]. In the heart, accumulation of intramyocellular triglycerides was also described in experimental models of obesity [7, 8]. Elevated circulating levels of fatty acids have been strongly associated to myocardial lipid accumulation [18, 19] and even to lipotoxicity in cardiomyocytes for oxidized LDL-cholesterol [24]. Moreover, presence of triglycerides and lipid metabolites has been related to cardiac lipotoxicity and cardiomyocyte apoptosis. Then, apoptotic cells were replaced by extracellular matrix that leads to cardiac remodeling and dysfunction [25]. A positive correlation between cardiac lipid accumulation and dysfunction has been described giving rise to the term lipotoxic cardiomyopathy associated with obesity [26]. Usually, early stages of cardiomyopathy include cardiac hypertrophy, intracellular lipid accumulation, fibrosis and diastolic dysfunction, which evolves to systolic dysfunction with reduced ejection fraction [27]. In the present study, there was no evaluation allowing to correlate heart function and accumulation of fatty acids per se. However, cardiomyocyte hypertrophy [13] and apoptosis, lipid deposit accumulation and fibrosis, as well as increased CPK were found, all together probably contributing to cardiac dysfunction associated to chronic high fat diet in *Psammomys obesus*. Plasma cardiospecific biomarkers (such as CK-MB) allowing the evaluation of heart function should be evaluated in further studies.

The present results suggest that obesity promoted myocardial lipid accumulation though fatty acid oversupply and chronic hyperlipidemia. Cardiac metabolism depends primarily on fatty acid utilization for oxidative phosphorylation and ATP generation [26]. Fatty acids enter the cell via cell membrane fatty acid transporters, such as CD36 and FABP [28]. In the present study, myocardial expressions of CD36 and FABP3 were increased, which suggest exaggerated entry of fatty acids into cardiac cell cytoplasm. Because of its rate-controlling role in myocardial fatty acid metabolism, CD36 has been implicated in dysregulated fatty acid and lipid metabolism in high fat diet-induced cardiomyopathy [29]. In the heart of obese rats, persistent relocation of fatty acid transporters CD36 and FABP from the cytosol to the cell membrane has been described [30–32] and associated to chronic elevation in cardiac fatty acid uptake [33]. Genetic studies also indicated a crucial role for FABP in the pathogenesis of metabolic cardiomyopathy [34]. Expression of CPT1B, a key mitochondrial enzyme implicated in β-oxidation, was also decreased in the present study. This strongly suggests decreased fatty acid oxidation, since mitochondrial transfer of fatty acid oxidation mainly depends on CPT activity [28]. Sixteen-week high fat diet resulted in an imbalance between fatty acid uptake and consumption, contributing to intracellular lipid accumulation, which probably leads to cardiac cell dysfunction and death [35] and subsequent cardiomyopathy and heart failure [36]. Indeed, lipotoxic cardiomyopathy has been identified as the major mechanism through which patients with metabolic syndrome and obesity develop cardiac hypertrophy and dysfunction [37].

Even if fatty acid oxidation contributes to the majority of ATP generation in the normal adult heart, cardiomyocytes present high and flexible metabolic ability to use glucose. In the present study, chronic high fat diet was associated with increased myocardial expression of GLUT1, while GLUT4 expression did not change. Both GLUT1 and GLUT4 are the main glucose transporters in the heart. Increased GLUT1expression suggests a shift from an oxidative to a glycolytic metabolism [38]. In high fat diet-fed transgenic mice overexpressing GLUT1 specifically in the heart, cardiac dysfunction associated to excessive lipid accumulation was observed [39]. Expression of PDK4, the major regulator of PDH, was also increased, suggesting constrained glucose oxidation in the heart after high fat diet [40]. This is consistent with previous study showing increased PK4 expression along with increased availability of fatty acids over glucose after high fat diet [41]. Cardiac PDH inhibition resulted in metabolic re-programming with reduced glucose oxidation and increased glycolysis [42], suggesting maladaptive metabolic remodeling after chronic high fat diet. These changes in energy supply and use are probably accompanied with reduced myocardial ATP production, due to the lower number of ATP molecules generated during glycolysis compared with fatty acid oxidation, which probably contributes to altered cardiac efficiency and function.

In the present study, cardiac expressions of different key energy sensors, including PPAR’s and AMPK was also evaluated. Expressions of PPAR-α and -γ were not altered in the heart after high fat diet. PPARs play key roles in regulating fatty acid metabolism in the heart [43, 44], through the induction of expression of molecules involved in fatty acid oxidation, such as CD36 and CPT1 [45, 46]. Myocardial PPAR-α overexpression mimicked lipotoxic cardiomyopathy [40], whereas PPAR-α knockdown attenuated it [47], suggesting its important role in lipotoxic cardiomyopathy pathogenesis. This apparent discrepancy with the present results has also been observed by others [8, 40, 48]. In diabetic mice, cardiac PPAR-α expression was not enhanced, while cardiac metabolism was altered [8, 40, 48]. This may be explained by the fact that alterations in cardiac metabolism could be independent of PPAR-α, or that PPAR-α activity could be enhanced independently of gene expression. AMPK detects intracellular ATP/AMP ratio and plays a pivotal role in intracellular adaptation to energy stress. Here, expression of AMPK was decreased after high fat diet. Dysregulated AMPK has also been described in the heart of high fat diet-fed mice [49, 50]. Cardiac AMPK activation has been involved in cardiac protection, accelerating ATP generation and attenuating ATP depletion, protecting against cardiac dysfunction and cardiomyocyte apoptosis [51, 52]. Inactivation of AMPK has been linked to the activation of apoptotic processes in cardiomyocytes [53], while AMPK activation reduced cardiomyocyte apoptosis and improved diabetic cardiomyopathy [54]. This is consistent with present results showing myocardial activation of apoptosis together with decreased AMPK expression after high fat diet.

UCP3 is a mitochondrial anion carrier protein with antioxidant properties known to stimulate fatty acid metabolism in muscle cells [55]. In high fat diet-fed animals, myocardial expression of UCP3 decreased. This is consistent with data showing either unchanged or decreased cardiac UCP3 levels in genetic models of obesity [56], despite increased mitochondrial uncoupling. In mice, genetic UCP3 deletion was associated to decreased myocardial ATP content [57], as well as worsening of cardiac function, increased cardiomyocyte death, and greater mortality after myocardial infarct [58]. Expression levels of insulin receptors was similar after a high fat diet. This is probably due to the fact that insulin resistance was not found in these animals. Increased expression of a heart failure marker BNP after a high fat diet was also shown in the present study. BNP is secreted by cardiomyocytes in response to stretching by increased blood volume. Increased expression of BNP precursor strongly suggests cardiac dysfunction associated to altered cardiac metabolism, as natriuretic peptide secretion has been shown to increase in proportion to the severity of cardiac dysfunction [59, 60]. However, further investigations are necessary to determine if these metabolic alterations are implicated in cardiac dysfunction in this experimental model.

### Study strength and limitation

*Psammomys obesus* is an original nutritionally controlled and genetically predetermined experimental model of metabolic syndrome, which is associated to sustained myocardial biological alterations [13]. However, heart function and plasma levels of cardiospecific biomarkers were not evaluated per se. This should be evaluated in further studies.

## Conclusions

In conclusion, after a sixteen-week high fat diet, the heart of *Psammomys obesus* showed signs of lipotoxic cardiomyopathy, characterized by metabolic remodeling and detrimental metabolic switch leading to lipid accumulation. This unique experimental model of nutritionally induced metabolic syndrome allowed us to myocardial impact of high fat diet in animals with a genetic predisposition to develop rapidly obesity, type 2 diabetes and metabolic syndrome. It may prove useful for the study of the mechanisms underlying the predisposition to develop insulin resistance and metabolic syndrome in humans who evolve from scarcity to abundant food intake.

## Data Availability

All relevant data generated and analyzed during the current study are included the present article.

## Abbreviations

AMPK: AMP-activated protein kinase
CD36: Fatty acid translocase
CPK: Creatine phosphokinase
CPT1B: Carnitine palmitoyltransferase-1B
FABP3: Fatty acid binding protein 3
HDL-c: High-density lipoprotein cholesterol
HPRT1: Hypoxanthine phosphoribosyl transferase 1
IRS1: Insulin receptor substrate1
IRS2: Insulin receptor substrate2
LDL-c: Low-density lipoprotein cholesterol
NPPB: Natriuretic peptide B
PDH: Pyruvate dehydrogenase
PDK4: Pyruvate dehydrogenase kinase 4
PPAR-α: Peroxisome proliferator activated receptors-alpha
PPAR-γ: Peroxisome proliferator activated receptor-gamma
Slc2a1 or GLUT1: Solute carrier family 2 members 1
Slc2a4 or GLUT4: Solute carrier family 2 members 4
TUNEL: Terminal Deoxynucleotidyl Transferase dUTP Nick-End Labeling
UCP3: Uncoupling protein 3
